# Dataset linking women's maternity care experiences with hospital environment and governance in Ireland

**DOI:** 10.1016/j.dib.2024.111168

**Published:** 2024-11-29

**Authors:** Adegboyega Ojo, Nina Rizun, Grace Walsh, Wojciech Przychodzen, Mona Isazad Mashinchi, Conor Foley, Daniela Rohde, Manohar Rao

**Affiliations:** a School of Public Policy and Administration, Carleton University, Ontario, Canada; b Gdansk University of Technology, Gdansk, Poland; c School of Business, Maynooth University, Kildare, Ireland; d CONNECT, the Science Foundation Ireland research centre for Future Networks and Communications, Ireland; e ESIC Business & Marketing School, Barcelona, Spain; f J.E. Cairnes School of Business and Economics, University of Galway, Ireland; g National Care Experience Programme, Health Information and Quality Authority, Cork, Ireland; h Insight Centre for Data Analytics, of Business, College of Science and Engineering, University of Galway, Ireland

**Keywords:** Maternity services, Trust in maternity services, Women-centred care, Maternity care standards, Trust in professional workforce, Governance

## Abstract

Many scholars argue that there is a deepening crisis of trust in healthcare systems. What is not contested is the centrality of public trust in building reputational value in healthcare organisations. However, there is a dearth of research focused on better understanding how trust in healthcare institutions, and the healthcare workforce, can be sustainably cultivated.

To enable the exploration of care-related factors within hospitals and their potential impacts on trust in healthcare workers, this dataset was created based on the 2020 National Maternity Experience Survey data. The survey data include responses to 68 structured, tick-box questions and three open-ended questions prepared with the participation of over 250 healthcare practitioners and experts, patients, as well as policymakers and researchers. The survey covers the full pathway of maternity care from antenatal care, through labour and birth, to postnatal care in the community. A total of 19 maternity hospitals and units participated in the survey which ran from February to April 2020, resulting in a total of 3204 women responses out of an eligible population of 6357. The survey data was extended with contextual information from a monitoring report on the National Maternity Services Standard published in 2020. The additional data includes compliance levels of maternity hospitals with established standards in four key areas including effective care support, safe care support, leadership governance and management, and workforce. This curated dataset can support investigations into a) the factors that determine overall women's care experience, b) factors contributing to building confidence and trust in the maternity care workforce among different groups of women, and c) how hospital environment, processes and governance impact both women's trust in maternity hospitals and their overall satisfaction.

Specifications Table

**Table:** 

Subject	Obstetrics, Midwifery and Womenʼs Health; Public Health and Health Policy; Decision Sciences (General); Management Science and Operations Research; Safety, Risk, Reliability and Quality.
Specific subject area	National maternity care services in Irish hospitals
Data format	Raw, Analysed and Filtered
Type of data	Table
Data collection	The dataset was created by integrating the 2020 National Maternity Experience Survey data collected by Ireland's National Care Experience Programme, with data curated from the 2020 National Maternity Services Standards Monitoring Report [1]. The model underpinning the maternity experience survey data is provided in [2]. The survey covers the entire maternity care pathway, from antenatal care, through labour and birth, to postnatal care in the community. The monitoring report covers compliance levels in four key dimensions of the Maternity Service Standards: effective care support, safe care support, leadership, governance and management, and workforce.
Data source location	Institution: Health Information Quality Authority City: Dublin Country: Ireland Latitude and longitude: 53.7798° N, 7.3055° W, Altitude: 118 msl
Data accessibility	Repository name: Open Science Framework Data identification number: 10.17605/OSF.IO/2AHGX Direct URL to data: https://osf.io/2ahgx/ Instructions for accessing these data: Click on the link above to access The questionnaire was administered, resulting in a raw dataset was created from the responses. Access to this raw dataset requires completing a data access request form and submitting it to the Health Information and Quality Authority. The authors of this publication were granted access to the raw dataset and from this, we curated the final “Maternity-Ireland-2020.xlsx” which is fully and openly available on the OSF platform (see https://osf.io/2ahgx/). The data access request form and data access request policy are also available at the above link.
Related research article	Ojo, A., Rizun, N., Walsh, G. S., Przychodzen, W., Mashinchi, M. I., Foley, C., & Rohde, D. (2023). Building confidence and trust in Ireland's National Maternity Services Workforce–What matters most and how? Health policy, 138, 104,947.

## 1 Value of the Data

• The value of the dataset lies in its potential to provide insights into how the quality of maternity care services may be influenced by factors such as (i) the levels of specialised care available for newborns based on their medical needs, i.e. (neonatal unit care levels,) and (ii) hospitals' compliance levels with national maternity service standards, such as the 2016 “National Standards for Safer and Better Maternity Services” [3] and the National Maternity Strategy [1]. Researchers interested in examining the alignment between service delivery and standards, as well as the implications of any disparities, are likely to find this dataset highly valuable [4]. Additionally, this dataset should be valuable for those interested in understanding the differences in what women consider important at various levels of neonatal care hospitals.
• Furthermore, this dataset affords a deeper exploration of how socioeconomic and demographic variables such as age, ethnicity and disability together with hospital environment related factors affect women's perceptions of the quality of maternity services [5]. Researchers also aiming to uncover how maternity care factors such as information provision, involvement in care, respect and dignity, and attention are impacted by compliance levels with care standards in different areas including workforce management will find the dataset valuable.
• The dataset has already enabled researchers to examine several key areas: the factors that determine overall women's care experience, the elements most critical to different groups of women in building confidence and trust in the maternity care workforce, and the impact of hospital environment such as neonatal care, processes and governance (e.g. compliance with standards) on women's trust in maternity hospitals and their overall satisfaction [[6], [7], [8]]. Further research could explore how demographic variables and hospital environment factors interact with perceived quality of care in various dimensions, providing a deeper understanding of the determinants of trust on healthcare workforce, and women's overall satisfaction.
• Finally, while this dataset provides a snapshot of one year's survey data, the survey is administered annually, enabling the potential for longitudinal research when integrated with future years’ publicly available survey data. This dataset is publicly available for download. Use and reuse are encouraged to promote research and studies aimed at improving maternity care services.

## 2 Background

This dataset is created to support research and analysis aimed at enhancing maternity health services. Primarily, it enables the identification of factors that influence women's confidence and trust in the professional workforce, as well as their overall satisfaction with maternity care. It will also support the provision of evidence on how compliance with maternity standards may (or may not) enhance maternity care service quality, trust in maternity services workforce and the overall satisfaction.

## 3 Data Description

This article describes the dataset including Excel file resource that is available as a supplementary file. The spreadsheet “*Maternity-Ireland-2020.xlsx*” in the repository contains the 12 sheets – 1) *Dataset* sheet containing the actual data with 72 columns covering core care constructs, basic metadata about maternity hospitals, and compliance levels of hospitals to maternity standards; 2) Constructs associated with maternity care professional workforce - Tab A; 3) Codes for hospital level of neonatal units – Tab B; 4) Codes for national maternity services standards monitoring report sections and corresponding national standard themes and inspection lines of enquiry - Tab C; 5) Codes for national maternity services standards description - Tab D; 6) Codes for level of compliance across the 19 maternity units and hospitals with seven of the national standards for safer better maternity services - Tab E; 7) Codes for counties - Tab F; 8) Codes for disability groups - Tab G; 9) Codes for age groups - Tab H; 10) Codes for ethnicity groups - Tab I; 11) Codes for hospital groups - Tab J; and 12) Codes for specific hospitals -Tab K.

The dataset is based on the first Irish National Maternity Experience Survey (NMES)^1^ carried out in 2020 as part of the National Care Experience Programme (NCEP). The base NMES data comprises 65 structured questions with predefined tick-box response options and three open-ended questions allowing free-text responses.^2^ Eligibility for participation was limited to women aged 16 or older who had given birth in October or November 2019 at one of the 19 maternity services included in the survey. Out of a potential participant pool of 6357, a total of 3206 women provided responses. Table 1 contains demographic details of the survey respondents.

**Table 1:** Demographics profile of respondents including their age, ethnicity, and disability groups (*N* = 3206).

Characteristic	Participants	
	Number	%
*Age group (years)*		
<25 years	155	4.84
25 to 29 years	451	14.08
30 to 34 years	1173	36.61
35 to 39 years	1145	35.74
40 and above	280	8.74
*Ethnic group*		
White	2951	92.0
Minority	255	8.0
*Disability group*		
Yes	582	18.16
No	2622	81.84
*Hospital Group*		
Saolta University Health Care Group	629	19.63
University Limerick Hospital Group	183	5.71
Ireland East Hospital Group	770	24.02
Dublin Midlands Hospital Group	424	13.23
RCSI Hospital Group	558	17.41
South/South West Hospital Group	623	19.44
Office of the Nursing and Midwifery Services Director	18	0.56
*Hospital Name*		
University Hospital Galway	118	3.68
Letterkenny University Hospital	122	3.81
Mayo University Hospital	157	4.90
Portiuncula University Hospital	106	3.31
Sligo University Hospital	126	3.93
University Maternity Hospital Limerick	183	5.71
National Maternity Hospital	361	11.26
Midland Regional Hospital Mullingar	151	4.71
St Lukes General Hospital	109	3.40
Wexford General Hospital	149	4.65
Coombe Women and Infants University Hospital	301	9.39
Midland Regional Hospital Portlaoise	123	3.84
Rotunda Hospital	335	10.45
Our Lady of Lourdes Hospital	105	3.28
Cavan General Hospital	118	3.68
Cork University Maternity Hospital	301	9.39
University Hospital Waterford	139	4.34
South Tipperary General Hospital	72	2.25
University Hospital Kerry	111	3.46
National Home Birth Services	18	0.56
*Hospital Neonatal Level*		
Level 1	1343	42.15
Level 2	545	17.11
Level 3	1298	40.74

The base NMES survey data consists of eight (8) sections. The first seven sections are designed to collect data on women's experiences in the following aspects of maternity care: (1) Care while pregnant or Antenatal Care - 17 questions; (2) Care during labour and birth - 10 questions; (3) Care in hospital after the birth - 9 questions; (4) Specialised care for the baby - 3 questions; (5) Feeding the baby - 5 questions; (6) Care at home after the birth - 14 questions; (7) Overall Care - 2 closed and 3 open-ended questions. The last part of the survey questionnaire is devoted to collecting the demographic data of the participant, such as (i) the woman's date of birth; (ii) the number of babies that a woman gave birth to before the current; (iii) ethnic group; (iv) country of residence (26 counties); and (v) whether the woman had mental health problems on a long-term basis (disability).

## 4 Experimental Design, Materials and Methods

The underpinning NMES survey data was provided to research team by the National Care Experience Programme in the context of a secondary analysis research project entitled *“Generating actionable insights from free-text care experience survey data using qualitative and computational text analysis*” [9]. The following steps were employed for extending and transforming the survey data to create the dataset - Maternity-Ireland-2020.xlsx [10].

**Data curation:** Our first activity entailed extending the received National Maternity Care Experience Survey data with the data curated from National Maternity Services Standards monitoring [1,3]. This comprised two steps, a) inclusion of the level of neonatal care provided by the hospitals in the survey data, and b) extending the dataset with information about the level of compliance of each hospital to different aspects of the maternity services standards.

*First Step –* We extended the survey data comprising 3206 rows with an additional column (labelled “Hospital Neonatal Unit”) that specifies for each row the level of *Neonatal care provided by the hospital in which the respondents received their maternity care*. Neonatal units range from local neonatal units (level 1) providing routine care to regional (level 2) units offering a broader range of care, to intensive care in tertiary neonatal units (level 3). According to the National Standards for Safer Better Maternity Services, level 1 neonatal units deliver routine neonatal care to term infants and special care to infants over 32 weeks' gestation. These units are staffed by consultant general paediatricians, non-consultant hospital doctors, and neonatal nurses. Level 2 neonatal units provide routine care to term infants, special care, high-dependency care, and short-term ventilation to infants over 27 weeks' gestation. These units follow Ireland's national model of neonatal care and are typically staffed with a combination of consultant neonatologists, paediatricians with a special interest in neonatology, non-consultant hospital doctors (NCHDs), and neonatal nurses. Level 3 neonatal units offer the full spectrum of specialized neonatal care for critically unwell-term and pre-term infants. These tertiary neonatal units are equipped with consultant neonatologists, non-consultant hospital doctors (NCHDs), and neonatal nurses.

*Second Step -* we enriched the survey data with 21 additional columns, that contains information about the degree of compliance of maternity hospitals to different aspects of maternity services standards as captured in standards *monitoring report*. Of the six major aspects covered in maternity services standard, four key aspects were selected based on their relevance: (1) Capacity and Capability Dimensions, that contains Leadership, Governance and Management - 11 items, and Workforce - 4 items; (2) Safety and Quality Dimension, that contains Effective Care - 8 items, and Support and Safe Care and Support - 5 items. *Tabs C & D of* ***Maternity-Ireland-2020.xlsx*** spreadsheet provides the descriptions of the four dimensions of the standards.

To specify the degree of compliance of hospitals maternity services with each of the standards, a 4-point scale is used in the reports, where - 4=“Compliant”; 3=“Substantially Compliant”; 2=“Partially Compliant”; 1= “non-compliant”. For each hospital in the survey data, matching was performed based on the data from National Maternity Services Standards monitoring report about degree of compliance of each of 19 hospitals maternity services with each of the standards, that is provided in the *Tab C* of the ***Maternity-Ireland-2020.xlsx*** spreadsheet.

**Data pre-processing and coding**: The women's experiences were measured using different scales including 3-, 4-, 5-, or 6-point scale in the survey data. For example, womenʼs experiences of getting satisfactory answers to questions during pregnancy is measured using a 5-point scale: 1=“Yes, always”; 2=“Yes, sometimes”; 3=“No”; 4=“I did not have any questions”; 5=“Don't know or can't remember”. Womenʼs feelings of involvement in decisions about their care was measured by the following 4-point scale: 1=“Yes, always”; 2=“Yes, sometimes”; 3=“No”; 4="Don't know or can't remember". These scales were recoded for more consistency in our dataset as follows. We assigned higher scores to greater positive answers, signifying good experience. For answers in which the respondents indicated ‘"Don't know or can't remember" or “not relevant” we coded the response empty values (assigned value −999). When respondent provided negative (i.e. No) to positively framed questions like *“Thinking about the care you received during your pregnancy, were your questions answered in a way that you could understand?”*, a low value is assigned signifying negative experience. Table 2 provides detailed information on the rules for recoding the original survey data. The indicators for which the number of non-empty values exceeded 20 % were removed from the dataset. As a result, 3204 rows were selected for building the final dataset.

**Table 2:** Inverse rules for recoding an indicatorʼs measurement scale.

Answers	Initial code	Reversed code
**5-point scale** *Question example:***"Thinking about the care you received *during your pregnancy*, were your questions answered in a way that you could understand?**"		
"Yes, always"	1	5
"Yes, sometimes"	2	4
"No"	3	1
“I did not have any questions”	4	3
“Don't know or can't remember”	5	−999 (as an empty value)
**4-point scale** *Question example:***Thinking about the care you received *during your labour and birth*, did you feel that you were involved in decisions about your care?"**		
“Yes, always”	1	4
“Yes, sometimes”	2	3
"No"	3	1
"Don't know or can't remember"	4	−999
**3-point scale** *Question example:***"Before you were discharged from hospital, were you told who to contact if you were worried about your health or your baby's health after you left hospital?"**		
"Yes "	1	1
"No"	2	0
"Don't know or can't remember"	3	−999
*6***-point scale** *Question example: "***Do you think your health care professionals did everything they could to help manage your pain during labour and birth?"**		
“Yes, definitely”	1	5
“Yes, to some extent”	2	4
“No”	3	1
“I did not need any help”	4	3
“Not relevant to my situation”	5	−999
“Don't know or can't remember”	6	−999

*Data Analysis*: Finally, 35 indicators that were relevant to our study on confidence and trust in professional workforce, were selected for our final dataset. These indicators were organized into 11 constructs. The process of constructing these categories involved three key steps: (a) grouping the indicators into thematic groups; (b) checking for the coherence of the emergent constructs; and (c) refining the constructs by removing indicators that did not fit well. As a result, we established a total of eleven distinct constructs (Table 3). Detailed descriptions of the constructs are provided in *Tab A of* the ***Maternity-Ireland-2020.xlsx*** [10].

**Table 3:** Description the constructs associated with professional workforce in maternity services.

Construct	# of indicators	Description
*Information*	8	Covers the provision of best available information by the healthcare service to enable women to make informed decisions about their care. These types of informational support may include advice and support on breastfeeding in addition to formula and bottle feeding; or information regarding the physical and mental changes prompted by maternity
*Involvement*	5	Covers the empowering women to participate in their own care decisions, as well as support in making decisions about infant feeding (breastfeeding/formula/bottle)
*Respect & Dignity*	3	covers ensuring women's right to be treated with respect, courtesy and consideration, such as through listening and addressing concerns
*Attention*	2	covers providing women with adequate care by trained and qualified health professionals who are involved in every step of care during delivery. For example, giving proper time and attention in the ward
*Confidence & Trust*	3	Covers supporting the development of a relationship of trust between a woman and her health care providers, for example by instilling confidence in new mothers by respecting their wishes; access to consistent aftercare service; the opportunity to build relationships, rapport, and trust with caregivers
*Communication*	8	Covers establishing and maintaining effective, adapted to the stage of women's care and circumstances, communication systems between all health workers, women and their families and these systems. This includes answering questions with clear explanations; communication after delivery, so that the mother is aware of any events
*Responsiveness*	2	Covers ensuring that maternity care providers are sensitive and responsive to the broad spectrum of circumstances that impact on the health and wellbeing of women and their babies. For example, feel monitored; and having access to needed help or to pain relief; ability access to support and care when additional assistance is needed; not feeling rushed or burdened by midwives
*Involvement of partner and/or companion*	1	Covers providing services and programs to encourage women to have a partner or person by their side to help them make informed decisions about their care, and to actively participate where possible, such as in decision-making processes in the ward and during delivery
*Pain management*	1	Covers providing all available options for pain relief during childbirth, as well as information about the features of their impact
*Personal circumstances*	1	Covers the provision of maternity care based on the woman's personal choice, combined with the assessed needs of the woman or her child, such as personalized care tailored to the woman's specific needs and circumstances; evidence that a woman's personal circumstances are taken into account when discussing her care
*Clarity of explanation*	1	Covers clearly explaining to women all examinations and procedures, providing answers to questions, discussing and explaining examination results and test results, including discussion of options and potential risks arising from decisions

**Fig. 1:**
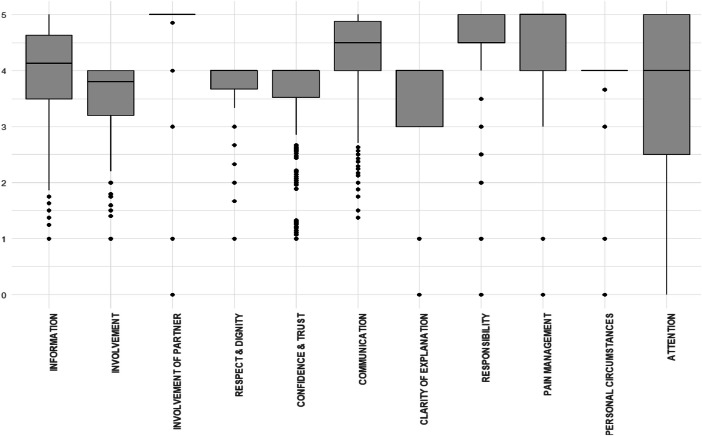
Boxplots of average women's experiences score distributions by constructs.

**Fig. 2:**
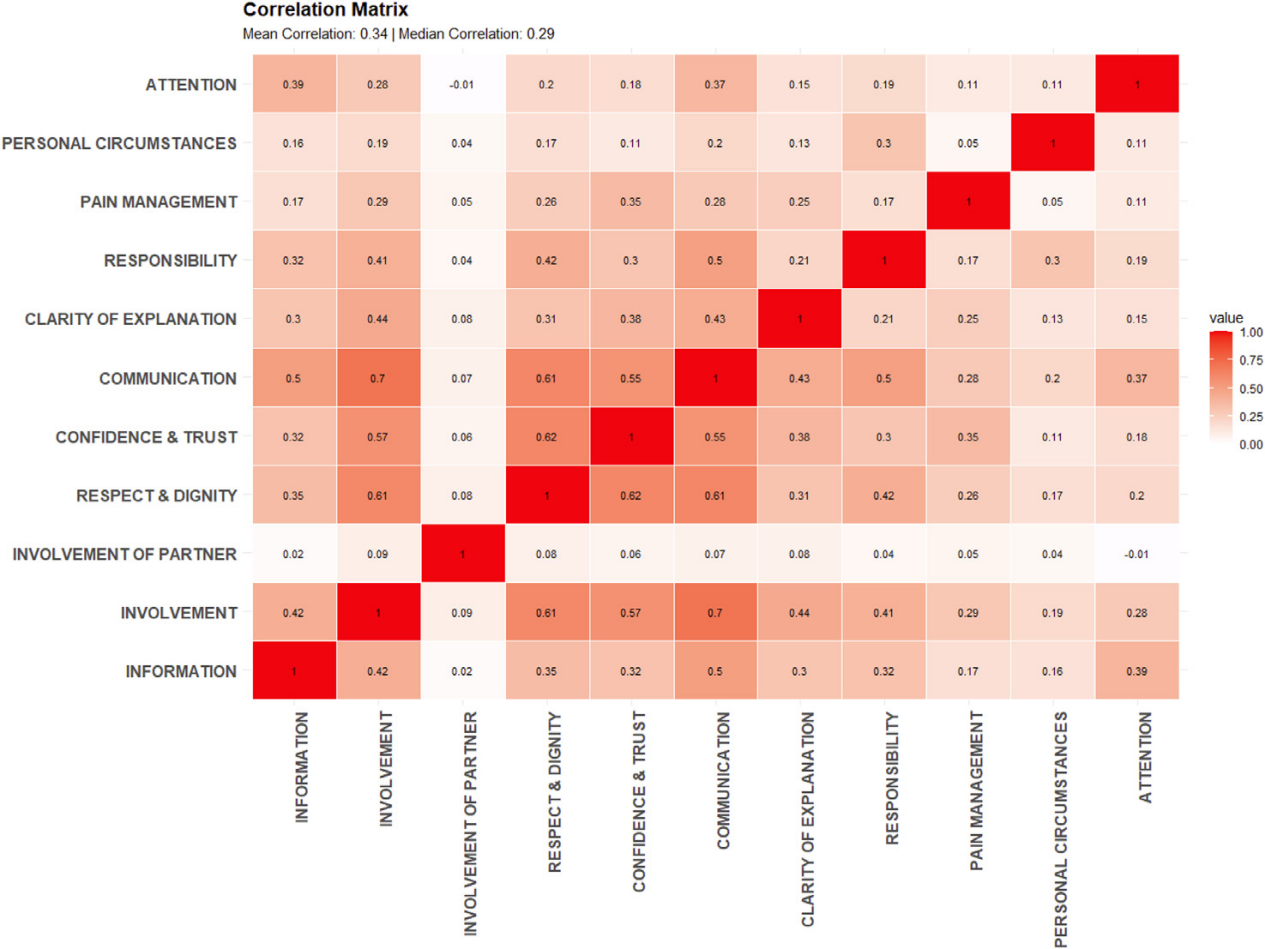
Correlation coefficients among constructs associated.

For each of the developed constructs, the average values of the included indicators were calculated. The average women's experiences score distributions for each of 11 constructs and overall Rating are reported in Boxplots (Fig. 1). The full descriptive statistics of our dataset constructs is provided in dataset repository at https://osf.io/2ahgx/. The correlation coefficients between among 11 determined constructs are presented in Fig. 2.

Thus, our final dataset (***Maternity-Ireland-2020.xlsx***) [10] contains 3204 rows and 71 columns, including (i) ResponseID and 35 initial dataset indicators; (ii) 11 constructs related to professional workforce; (iii) overall *Rating scores*; and (iv) selected metadata from the *National Maternity Care Experience Survey*, such as County, Disability Groups, Age Groups, Ethnicity Groups, Hospital, and Hospital Group; and (iv) *National Maternity Services Standards monitoring-related metadata*, including Hospital Neonatal level and the degree of compliance of Hospitals’ maternity services HIQA's monitoring programme Standards.

## Limitations

The dataset was created using a secondary dataset and freely available service standards monitoring reports. Second, our dataset did not allow us to explore how determinants vary with different socio-demographic categories like ethnicity. There is relatively small proportion of ethnic and minority groups in Ireland using maternity services (under 6 %).

Concerning the original, raw data upon which this dataset is based there are various limitations. The first clear shortcoming with a patient satisfaction survey is response bias, ensuring a strong response rate and comparing results across hospitals are two potential methods of addressing response bias [11]. Positivity bias is another issue present in customer satisfaction surveys whereby self-reports of satisfaction possess distributions that are negatively skewed and exhibit a positivity bias [12]. This is a key issue within the health services domain [13]. According to [14] patient satisfaction survey scores tend to be overly positive due to perceived social desirability of positive scoring. When the majority of scores are favourable, focusing on extreme responses may create more discrimination or variability in the scores [15].

## Ethics Statement

This study involves secondary analysis of anonymised quantitative and qualitative data from the National Maternity Experience Survey. Ethical approval for the National Maternity Experience Survey was granted by the Royal College of Physicians in Ireland (RCPI) on 28 January 2019.

## CRediT Author Statement

**Adegboyega Ojo** – Conceptualization, Methodology, Supervision, Funding acquisition, Writing - Review & Editing, Visualization. **Nina Rizun** – Methodology, Validation, Data Curation, Formal analysis, Investigation, Visualization. **Grace Walsh** - Writing - Original Draft, Writing - Review & Editing. **Wojciech Przychodzen** - Writing - Review & Editing, Investigation. **Mona Isazad Mashinchi** - Methodology, Formal analysis, Data Curation, Project administration. **Conor Foley** - Validation, Resources, Writing - Review & Editing. **Daniela Rohde** – Validation, Resources, Writing - Review & Editing. **Manohar Rao** – Data Management, Data Curation.

## Data Availability

OSFMaternity-Ireland-2020.xlsx (Original data).

## Footnotes

1 National Maternity Experience Survey.

2 Survey model and methodology.
